# A SCOPING REVIEW OF PARTICIPANT MOTIVATIONS, BENEFITS, AND BURDENS IN END-OF-LIFE RESEARCH PARTICIPATION

**DOI:** 10.1093/geroni/igae098.2378

**Published:** 2024-12-31

**Authors:** Cara Wallace, Stephanie Wladkowski, Ruaa Al Juboori, Anna Wingo, Rebecca Hyde

**Affiliations:** Saint Louis University, St. Louis, Missouri, United States; Bowling Green State University, College of Health & Human Services, Bowling Green, Ohio, United States; University of Mississippi, Oxford, Mississippi, United States; Saint Louis University, St. Louis, Missouri, United States; Saint Louis University, St. Louis, Missouri, United States

## Abstract

Hospice clinicians are often hesitant to ask patients and their families/caregivers to participate in research, fearing it is too intrusive during a sensitive and vulnerable time. Further, there is a lack of research, highlighting a gap in knowledge of patient and family experience during hospice and palliative care while it also inhibits the understanding of the benefits and burdens of participation in end-of-life research. A scoping review aimed at understanding the motivating factors, benefits, and burdens of participation in research for patients with a terminal illness and their families/caregivers was conducted. Partnering with a librarian scientist, we searched PscyInfo, CINAHL, PubMed, and Scopus for studies published between 2015 and 2024 using the following search terms: “palliative care,” “terminal illness,” or “hospice” AND “subject burden,” research burden,” “benefit,” or “motivation.” 3,431 unique articles were identified and reviewed, with 24 studies meeting inclusion criteria. Results from these 24 studies were mapped out using the Preferred Reporting Items for Systematic reviews and Meta-Analyses extension for Scoping Reviews (PRISMA-ScR) guidelines. Many participants reported greater benefits than burdens, even reporting great value in participation when a burden was experienced. Individuals also reported that participation in a study provided an additional outlet for support, and feeling a socioemotional altruistic benefit even with no direct medical benefit as a study participant. For clinicians working in end-of-life care, connecting patients and caregivers to end-of-life research may not only be beneficial to contribute to literature as well as an additional source of ongoing support for patients and their caregivers.

